# Efficacy of Colistin and Its Combination With Rifampin *in Vitro* and in Experimental Models of Infection Caused by Carbapenemase-Producing Clinical Isolates of *Klebsiella pneumoniae*

**DOI:** 10.3389/fmicb.2018.00912

**Published:** 2018-05-15

**Authors:** María E. Pachón-Ibáñez, Gema Labrador-Herrera, Tania Cebrero-Cangueiro, Caridad Díaz, Younes Smani, José P. del Palacio, Jesús Rodríguez-Baño, Alvaro Pascual, Jerónimo Pachón, M. Carmen Conejo

**Affiliations:** ^1^Clinical Unit of Infectious Diseases, Microbiology, and Preventive Medicine, Institute of Biomedicine of Seville, University Hospital Virgen del Rocío/CSIC/University of Seville, Seville, Spain; ^2^Fundacion Centro de Excelencia en Investigación de Medicamentos Innovadores en Andalucía, MEDINA Foundation, Granada, Spain; ^3^Clinical Unit of Infectious Diseases, Microbiology, and Preventive Medicine, Institute of Biomedicine of Seville, University Hospital Virgen de Macarena/CSIC/University of Seville, Seville, Spain; ^4^Department of Medicine, University of Seville, Seville, Spain; ^5^Department of Microbiology, University of Seville, Seville, Spain

**Keywords:** *Klebsiella pneumoniae*, animal models, carbapenemase producers, colistin, rifampin

## Abstract

Despite the relevance of carbapenemase-producing *Klebsiella pneumoniae* (CP-Kp) infections there are a scarce number of studies to evaluate *in vivo* the efficacy of combinations therapies. The bactericidal activity of colistin, rifampin, and its combination was studied (time–kill curves) against four clonally unrelated clinical isolates of CP-Kp, producing VIM-1, VIM-1 plus DHA-1(acquired AmpC β-lactamase), OXA-48 plus CTX-M-15 (extended spectrum β-lactamase) and KPC-3, respectively, with colistin MICs of 0.5, 64, 0.5, and 32 mg/L, respectively. The efficacies of antimicrobials in monotherapy and in combination were tested in a murine peritoneal sepsis model, against all the CP-Kp. Their efficacies were tested in the pneumonia model against the OXA-48 plus CTX-M-15 producers. The development of colistin-resistance was analyzed for the colistin-susceptible strains *in vitro* and *in vivo*. *In vitro*, colistin plus rifampin was synergistic against all the strains at 24 h. *In vivo*, compared to the controls, rifampin alone reduced tissue bacterial concentrations against VIM-1 and OXA-48 plus CTX-M-15 strains; CMS plus rifampin reduced tissue bacterial concentrations of these two CP-Kp and of the KPC-3 strain. Rifampin and the combination increased the survival against the KPC-3 strain; in the pneumonia model, the combination also improved the survival. No resistant mutants appeared with the combination. In conclusion, CMS plus rifampin had a low and heterogeneous efficacy in the treatment of severe peritoneal sepsis model due to CP-Kp producing different carbapenemases, increasing survival only against the KPC-3 strain. The combination showed efficacy in the less severe pneumonia model. The combination prevented *in vitro* and *in vivo* the development of colistin resistant mutants.

## Introduction

Carbapenem-resistant *Klebsiella pneumoniae* strains are spreading worldwide, representing an urgent threat to public health, as stressed by the Center for Disease Control and Prevention (CDC) of United States, the European Centre for Disease Prevention and Control (ECDC) and the World Health Organization (WHO). The rapid spread, mostly in hospital settings, is transforming many common health care-associated complications into infections that are sometimes untreatable with the currently available antimicrobials (Nordmann et al., 2012).

The carbapenem resistance *in K. pneumoniae* is mainly due to the production of acquired carbapenemases (Falagas et al., 2014). The most important carbapenemases found in this species may belong to the Ambler classes A (mainly KPC), B (the most frequent are VIM and IMP) and D (OXA-48-like enzymes). Invasive infections by isolates producing VIM and KPC are associated with high death rates (Tzouvelekis et al., 2012). The information about infections caused by OXA-48-producers is scarce, mostly because of its difficult identification (Nordmann et al., 2011; Canton et al., 2012; Tzouvelekis et al., 2012). Nevertheless, OXA-48 is the most frequent carbapenemase produced by Enterobacteriaceae isolated in many European countries (Canton et al., 2012; Palacios-Baena et al., 2016; De Laveleye et al., 2017).

Although the hydrolysis spectrum of these enzymes may vary, they hydrolyze most beta-lactams, including carbapenems. Moreover, carbapenemase producers often show co-resistance to other antimicrobial agents, leaving very few treatment options, such as tigecycline, colistin, and some aminoglycosides (de Oliveira et al., 2015). So, in an attempt to improve the poor clinical efficacy of the available drugs, combination therapy is often used as definitive therapy for infections caused by carbapenemase-producing *K. pneumoniae* (CP-Kp). To date, recommendations are based on few retrospective clinical studies and *in vitro* studies (Tzouvelekis et al., 2012). In addition, a scarce number of *in vivo* studies have assessed the efficacy of antimicrobial combinations against CP-Kp (Tzouvelekis et al., 2012). Clinical studies have reported favorable outcomes for patients treated with combinations of colistin and a carbapenem, tigecycline, fosfomycin, or an aminoglycoside (Michalopoulos et al., 2010; Lee and Burgess, 2012; Munoz-Price et al., 2013; Daikos et al., 2014). However, little data exists on which combination therapy is superior.

Several studies have reported synergistic activity of colistin and rifampin against colistin-resistant and colistin-susceptible KPC-producing *K. pneumoniae* clinical strains, using the checkerboard method. Similarly, studies based on time-kill experiments detected synergy with this combination against colistin-resistant KPC or NDM producers clinical strains, but no synergistic effect against VIM producers. To our knowledge, there is no data regarding the efficacy of this combination against *K. pneumoniae* producing OXA-48, nor *in vivo* studies to validate the *in vitro* results previously mentioned (Elemam et al., 2010; Tascini et al., 2013; Nastro et al., 2014; Tangden et al., 2014). Thus, the aim of this study was to evaluate the *in vitro* and *in vivo* efficacy of CMS plus rifampin against CP-Kp clinical strains producing different carbapenemases.

## Materials and Methods

### Bacterial Strains, Beta-lactamase Characterization and Molecular Typing

Four genetically unrelated clinical isolates of CP-Kp were studied: Kp07, a VIM-1 ST 1603 clone producer (Miro et al., 2013); Kp21, which co-produced VIM-1 and the acquired AmpC type beta-lactamase DHA-1 ST 11 clone (Miro et al., 2013); Kp28, co-producing OXA-48 ST11 clone and the extended spectrum beta-lactamase (ESBL) CTX-M-15 (Oteo et al., 2015); and Kp29, co-producing KPC-3 ST512 clone with the broad spectrum beta-lactamases TEM-1 and SHV-11 (Lopez-Cerero et al., 2014), thereinafter VIM-1, VIM-1/DHA-1, OXA-48 plus CTX-M-15, and KPC-3 producers, respectively. Identification of these isolates was confirmed by a Microflex LT-MALDI Biotyper mass spectrometer (Bruker Daltonics GmbH, Bremen, Germany). The presence of carbapenemase genes, and genes coding for other beta-lactamases was confirmed by PCR and sequencing as described previously. The absence of genetic relation among the isolates was confirmed by PFGE analysis of chromosomal restriction fragments obtained after *Xba*I cleavage following the criteria of Tenover et al. (1995). Two of the strains, KPC-3 and VIM-1 (DHA-1) were multidrug-resistant while the other two were not. The antibiotic susceptibility profiles are included in the Supplementary Information.

### Antimicrobials

For the *in vitro* assays, antimicrobials, colistin sulfate salt and rifampin, were used as standard laboratory powders (Sigma-Aldrich, Madrid, Spain). For *in vivo* experiments, clinical formulations were used: colistimethate sodium (CMS) (Genéricos Españoles S.A., Madrid, Spain) and rifampin (Sanofi-Aventis, Madrid, Spain).

### *In Vitro* Studies

#### Antimicrobial Susceptibility Testing

MICs of antibiotics were determined by broth microdilution as recommended by the Clinical and Laboratory Standards Institute [CLSI] (2012), using Mueller Hinton broth II (MHB) (Becton Dickinson & Co., Sparks, MD, United States) and agar dilution method for fosfomycin. MIC results were interpreted according to the European Committee on Antimicrobial Susceptibility Testing [EUCAST] (2016)^1^ breakpoints. Studies were performed in triplicate.

#### Time-Kill Curves

The concentrations of colistin used for susceptible strains corresponded to the MIC value obtained by microdilution, whereas the concentration used for resistant strains was 2 mg/L, the susceptibility breakpoint recommended by European Committee on Antimicrobial Susceptibility Testing [EUCAST] (2016). Rifampin was used at a fixed concentration of 2 mg/L. Experiments were carried out with a starting inoculum of 1 × 10^6^ cfu/mL and the antibiotics alone or in combination. Tubes were incubated at 37°C, with shaking, and samples were taken at 0, 1, 3, 6, and 24 h, serially diluted and plated using a spiral platter (Eddy Jet, IUL S.A., Barcelona, Spain). Bacterial colonies were counted using an automatic counter (Flash & Go, IUL S.A., Barcelona, Spain) (Pournaras et al., 2011; Souli et al., 2011). Experiments were performed three times on separate occasions. Synergy was defined as a decrease ≥ 2 log_10_ cfu/mL for the antimicrobial combination compared with the most active single agent. Bactericidal activities of single antibiotics or combination were defined as a decrease ≥ 3 log_10_ cfu/mL from the starting inoculum (Souli et al., 2009). Studies were performed in triplicate.

### Animals

Immunocompetent C57BL/6 female mice weighing approximately 20 g (Production and Experimentation Animal Center, University of Seville, Seville, Spain) were used; they had a sanitary status of murine pathogen free and were assessed for genetic authenticity. Mice were housed in an individually ventilated cage system under specific pathogen-free conditions, and water and food supplied *ad libitum*. This study was carried out following the recommendations in the Guide for the Care and Use of Laboratory Animals (Souli et al., 2011). Experiments were approved by the Committee on the Ethics of Animal Experiments of the University Hospital Virgen Macarena, Seville, Spain (CI 1961). All procedures were performed under sodium thiopental (B. Braun Medical S.A., Spain) anesthesia, and all efforts were made to minimize suffering.

### Pharmacokinetic/Pharmacodynamic Analysis

Serum antibiotic concentrations were determined in healthy mice after a single intraperitoneal (ip) administration of CMS (20 mg/kg) or rifampin (25 mg/kg). In sets of three anesthetized mice, blood samples from the periorbital plexus were obtained at different time points after the administration of CMS and rifampin. Blood samples were immediately centrifuged (4500 rpm, 15 min at 4°C), and serum samples stored at -80°C until the analysis. Both serum free and total antibiotic concentrations were measured using HPLC-tandem mass spectrometry (LC-MS/MS) (Waters et al., 2008; Gobin et al., 2010). Free fractions of the drugs were calculated as described previously (Waters et al., 2008; Gonzalez et al., 2013; Cheah et al., 2015).

Maximum concentration of drug in serum (C_max_), elimination half-life (*t*_1/2_), area under the concentration-time curve from 0 to 24 h (AUC_0-24_), free AUC_0-24_ (*f*AUC_0-24_), AUC_0-24_/MIC and *f*AUC_0-24_/MIC ratios were calculated using the PKSOLVER program (Zhang et al., 2010). The pharmacodynamic targets to assess the efficacy were *f*AUC_0-24_/MIC and AUC_0-24_/MIC for CMS and rifampin, respectively.

### Experimental Models

#### Peritoneal Sepsis Model

A previously described murine peritoneal sepsis model was used (Parra Millan et al., 2016). Briefly, groups of un-anesthetized C57BL/6 mice were infected by ip injection of 0.5 mL of the Minimal Lethal Dose (MLD) of each strain, corresponding to (log_10_ cfu/mL): 8.97, 8.94 9.35, and 8.38 for VIM-1, VIM-1/DHA-1, OXA-48 plus CTX-M-15, and KPC-3 strains, respectively. Treatments were initiated 4 h post-inoculation. For each one of the strains, mice were randomly included into four different therapeutic groups: (1) controls (untreated), (2) CMS, 20 mg/kg/8 h/ip, (3) rifampin, 25 mg/kg/6 h/ip, and (4) CMS plus rifampin, using the same dosing schedule as in monotherapy. The sample size for the combination groups and CMS monotherapy for the colistin-susceptible strains (VIM-1 and OXA-48 plus CTX-M-15) was 15 mice; nevertheless, for the monotherapies in case of resistance to colistin (VIM-1/DHA-1 and KPC-3) and for rifampin, the sample size was 10 to accomplish the 3Rs (Replacement, Reduction and Refinement) rules for performing animal research. Afterward, mice were treated and monitored for 72 h. The antimicrobial dosages were based on the PK/PD data, and their proven efficacy, alone and in combination, in previous experimental murine models of infection (Pachon-Ibanez et al., 2010). Samples were extracted and processed immediately after the death of mice; the survivor mice were sacrificed (sodium thiopental) at the 72 h. Aseptic thoracotomies were carried out, and blood samples were obtained for qualitative blood cultures; results were expressed as positive (≥1 cfu present in the plate) or negative. Spleens were aseptically extracted, weighed, and homogenized in sterile saline (Stomacher 80; Tekmar Co., Cincinnati, OH, United States) before quantitative cultures (log_10_ cfu/g) in Columbia agar with 5% sheep blood plates.

### Pneumonia Model

A previously characterized murine pneumonia model was used (Pachon-Ibanez et al., 2010; Docobo-Perez et al., 2012; Parra Millan et al., 2016) with the OXA-48 plus CTX-M-15 strain, as OXA-48 is the most prevalent carbapenemase in Spain nowadays (Palacios-Baena et al., 2016). Anesthetized mice were infected intratracheally, using 50 μl of a final inoculum of 8.18 log_10_ cfu/mL. Therapies were initiated 4 h post-infection and treatment groups and dosages were the same as in the peritoneal sepsis model. Mice were treated and monitored over 72 h. After death or sacrifice of the mice at 72 h, blood and lungs samples were aseptically obtained and processed as detailed above.

### *In Vitro* and *in Vivo* Selection of Colistin-Resistant and Rifampin-Resistant Mutants

Up to 10 colonies, of colistin-susceptible strains exposed during 24 h to colistin at the MIC, alone or in combination with rifampin, in time-kill curves, and from controls (non-exposed to the antimicrobials), were sub-cultured twice in antimicrobial free medium and frozen at -80°C until MIC testing.

In the experimental murine pneumonia model, after processing the lungs as described above, the remaining homogenized tissue was vortexed and centrifuged, and the pellet was resuspended in 2 mL of sterile saline. All the volume was spread on agar and incubated for 48 h at 37°C. A maximum of ten colonies recovered from each lung were selected, sub cultured in antibiotic free medium twice and frozen at -80°C until MIC testing.

MICs determinations of colistin and rifampin were carried out in triplicate.

### Statistical Analysis

Mortality and bacteremia rates are expressed as percentages and bacterial tissue concentrations (log_10_ cfu/g) as means ±*SD*. The two-tailed Fisher’s test, analysis of variance (ANOVA), and the Dunnet and Tukey *post hoc* tests were used. A *P* < 0.05 was considered significant. The SPSS v22.0 statistical package was used (SPSS Inc).

## Results

### *In Vitro* Results

#### Antimicrobial Susceptibility Testing

MICs of each antibiotic for the four clinical isolates are shown in **Table 1**. Two strains were resistant to colistin (VIM-1/DHA-1 and KPC-3 producers).

**Table 1:** MICs of colistin and rifampin for the four carbapenemase-producing *K. pneumoniae* clinical strains.

Strains	MIC (mg/L) [SIR]	
	Colistin	Rifampin
VIM-1	0.5 [^∗^]	32 [^∗∗∗^]
VIM-1/DHA-1	64 [^∗∗^]	>128 [^∗∗∗^]
OXA-48/CTX-M-15	0.5 [^∗^]	32 [^∗∗∗^]
KPC-3	32 [^∗∗^]	64 [^∗∗∗^]

*^∗^Susceptible, MIC ≤ 2 mg/L. ^∗∗^Resistant, MIC > 2 mg/L. ^∗∗∗^Breakpoints are not defined (intrinsically resistant).*

#### Time-Kill Curves

**FIGURE 1:**
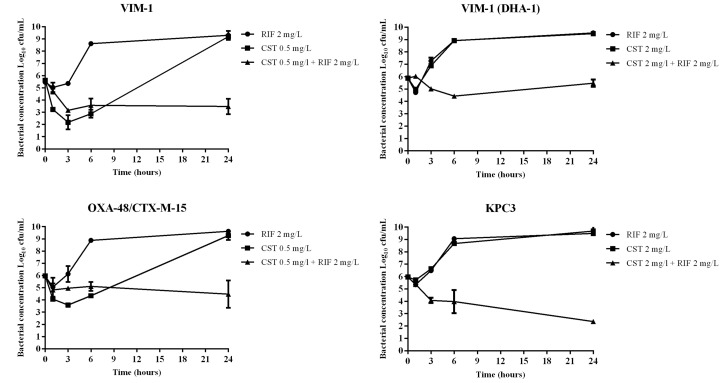
Time-kill curves for colistin (CST) and rifampin (RIF) alone and in combination against four clinical strains of carbapenemase producing strains (VIM-1, VIM-1/DHA-1, OXA-48/CTX-M-15 and KPC3). The CST concentration used for colistin-susceptible strains corresponded to the value of their MICs (colistin 0.5 mg/L for VIM-1 and OXA-48/CTX-M-15); for colistin-resistant isolates the concentration of CST was that corresponding to the susceptibility breakpoint recommended by EUCAST (colistin 2 mg/L for VIM-1/DHA-1 and KPC-3). The RIF concentration used for all strains was 2 mg/L. RIF, filled circles; CST, filled squares; combination of both antimicrobials, filled triangles.

The results are shown in **Figure 1**. Colistin was bactericidal at 3 h against the VIM-1 producer, but an important regrowth at 24 h. In addition, colistin reduced bacterial concentration (2.5 log_10_ decrease in cfu/mL) of the OXA-48 plus CTX-M-15-producing isolate, again with an important regrowth at 24 h. Colistin did not display any bactericidal activity against the colistin-resistant isolates (VIM-1/DHA-1 and KPC-3 producers). Rifampin did not show bactericidal activity against any strain. The combination was synergistic against all the strains at 24 h, but achieving only a bacteriostatic effect against the VIM-1, VIM-1/DHA-1, and OXA-48 plus CTX-M-15 isolates; on the contrary, the combination was bactericidal against the KPC-3 isolate.

### Pharmacokinetics and Pharmacodynamics

Pharmacokinetic parameters of each antimicrobial are shown in **Table 2**. Pharmacodynamics profiles are shown in **Tables 3**, **4**.

**Table 2:** Pharmacokinetic profiles of CMS and rifampin in mice serum.

Antimicrobial (dose [mg/kg], route of administration)	Drug	C_max_ (mg/L)	*t*_1/2_ (h)	AUC_0-24_ (mg⋅h/L)
CST (20, ip)	*t*CST	2.87	3.82	13.65
	*f*CST	0.24	3.82	1.15
RIF (25, ip)	*t*RIF	72.58	19.33	1103.58
	*f*RIF	2.90	19.33	44.14

*CST, colistin; RIF, rifampin; ip, intraperitoneal; tCST, total colistin; fCST, free colistin; tRIF, total rifampin; fRIF, free rifampin; ip., intraperitoneal; C_max_, maximum concentration of drug in serum; t_1/2,_ half-life; AUC_0-24,_ area under the concentration-time curve from 0 to 24 h; fAUC_0-24_, the unbound fraction AUC_0-24_.*

**Table 3:** *In vivo* efficacy and pharmacodynamics of CMS and rifampin, alone and in combination, for the experimental peritoneal sepsis model.

Strain	Treatment group	*n*	Log_10_ cfu/g spleen (mean ±*SD*)	Bacteremia (%)	Mice mortality (%)	AUC_0-24_/MIC^∗^	*f*AUC_0-24_/MIC^∗^
VIM-1	CTL	9	8.92 ± 0.46	100	100	NA	NA
	CST	15	8.60 ± 0.30	100	93.33	NA	2.30
	RIF	8	7.14 ± 0.48^a^	100	100	34.50	NA
	CST + RIF	15	6.01 ± 1.77^a^	53.33^b^	93.33	NA	NA
VIM-1/DHA-1	CTL	10	9.46 ± 0.32	100	100	NA	NA
	CST	6	9.54 ± 0.34	100	100	NA	0.04
	RIF	8	9.68 ± 0.09	100	100	8.60	NA
	CST+RIF	15	7.64 ± 3.02	73.33	73.33	NA	NA
OXA-48 plus CTX- M-15	CTL	10	9.56 ± 0.47	100	100	NA	NA
	CST	15	9.52 ± 0.55	100	100	NA	2.30
	RIF	10	8.24 ± 0.76^c^	100	100	34.50	NA
	CST+RIF	15	7.06 ± 2.50^c^	87.67	73.33	NA	NA
KPC-3	CTL	10	10.19 ± 0.29	100	100	NA	NA
	CST	8	9.19 ± 1.85	100	75	NA	0.02
	RIF	9	7.20 ± 2.96	88.88	66.67^d^	17.20	NA
	CST+RIF	15	6.76 ± 2.48^d^	77	40^d^	NA	NA

*CTL, control (no antimicrobial treatment); CST, colistin; RIF, rifampin; cfu, colony-forming unit; AUC_0-24_, area under the concentration-time curve from 0 to 24 h; fAUC_0-24_, the unbound fraction AUC_0-24_. ^∗^PD parameters were determined in healthy mice after CMS or rifampin injection. ^a^P ≤ 0.02 compared to CTL and CST groups. ^b^P < 0.05 compared to CTL, CST, and RIF groups. ^c^P < 0.05 compared to CTL and CST groups. ^d^P < 0.05 compared to CTL group.*

**Table 4:** *In vivo* efficacy and pharmacodynamics of CMS and rifampin, alone and in combination, for the experimental pneumonia model.

Strain	Treatment group	*n*	Log_10_ cfu/g lung (mean ±*SD*)	Bacteremia (%)	Mice mortality (%)	AUC_0-24_/MIC^∗^	*f*AUC_0-24_/MIC^∗^
OXA-48 plus CTX- M-15	CTL	15	8.05 ± 2.36	53.30	40	NA	NA
	CST	15	5.14 ± 3.78	40	26.70	NA	2.30
	RIF	15	3.07 ± 1.63^a^	20	13.30	34.50	NA
	CST+RIF	16	2.99 ± 1.36^a^	31.25	6.26^a^	NA	NA

*CTL, control (no antimicrobial treatment); CST, colistin; RIF, rifampin; cfu, colony-forming unit, AUC_0-24_, area under the concentration-time curve from 0 to 24 h; fAUC_0-24_, the unbound fraction AUC_0-24_. ^∗^PD parameters were determined in healthy mice after CMS or rifampin injection. ^a^P < 0.05 compared to CTL group.*

### *In Vivo* Results: Peritoneal Sepsis Model

The efficacies of the antimicrobials are shown in **Table 3**. Mortality, bacterial clearance on spleen and bacteremia, are analyzed immediately after the death of mice or at the end of the experiment (72 h of treatment).

#### Mortality

Mortality in all control groups (non-treated) was 100% within the first 24 h post-infection. Rifampin and its combination with CMS reduced mortality in animals infected with the KPC-3 strain to a 66.67% and a 40%, respectively, but not CMS alone. There was no reduction of mortality with any treatment when the peritoneal sepsis was produced by the other strains.

#### Bacterial Clearance From Spleen

Rifampin alone improved significantly the clearance of bacteria from spleen (CFU/g of tissue) compared with the control and the CMS groups in mice infected with either of the two colistin-susceptible strains producing VIM-1 (7.14 ± 0.48 vs. 8.92 ± 0.46 and 7.14 ± 0.48 vs. 8.60 ± 0.30, respectively) or OXA-48 plus CTX-M-15 (8.24 ± 0.76 vs. 9.56 ± 0.47, and 8.24 ± 0.76 vs. 9.52 ± 0.55, respectively). CMS plus rifampin reduced the bacterial concentration compared with the controls for both colistin-susceptible strains, producing VIM-1 or OXA-48 plus CTX-M-15 and for the colistin-resistant strain producing KPC-3, (6.01 ± 1.77 vs. 8.92 ± 0.46, 7.06 ± 2.50 vs. 9.56 ± 0.47, and 6.76 ± 2.48 vs. 10.19 ± 0.29, respectively). The combination was also better than CMS alone for both colistin-susceptible strains (6.01 ± 1.77 vs. 8.60 ± 0.30, 7.06 ± 2.50 vs. 9.52 ± 0.55, respectively).

#### Bacteremia

CMS plus rifampin showed the best effect among all the treatment groups in sterilizing blood cultures of mice infected with any of the four strains. Nevertheless, this reduction was only significant in mice infected with the VIM-1 producer (53.33% vs. 100%).

### Pneumonia Model Results

**Table 4** summarizes the results for the pneumonia model.

#### Mortality

The severity of this model was lower, with a mortality of 40% at the end of the experiment (72 h) using the MLD in the control group. Only the combination therapy decreased significantly mortality compared with the control group (6.26% vs. 40%; *P* < 0.05).

#### Bacterial Clearance From Lungs

Rifampin alone and its combination with CMS decreased significantly bacterial lung concentration compared with the control (3.07 ± 1.63 vs. 8.05 ± 2.36, and 2.99 ± 1.36 vs. 8.05 ± 2.36, respectively).

#### Bacteremia

None of the antimicrobial treatments achieved a significant reduction of the bacteremia in comparison with the control group.

### *In Vitro* and *in Vivo* Selection of Colistin-Resistant Mutants and Rifampin-Resistant Mutants

Colistin MICs of colonies recovered from susceptible strains (producing VIM-1 or OXA-48 plus CTX-M-15) after exposure at the MIC during 24 h in time-kill curves increased from 0.5 mg/L to > 8 mg/L. No mutants resistant to colistin were detected when these strains were exposed to the combination of colistin plus rifampin.

The MIC of rifampin for the colonies recovered from the lungs of mice challenged with OXA-48 plus CTX-M-15 producer and treated with rifampin alone increased from 16 to > 256 mg/L (16-folds) and the MIC of colistin from the colonies recovered from the lungs of mice treateted with CMS as monotherapy increased from 1 to > 32 mg/L (32-folds). Nevertheless, the MICs of rifampin and colistin remained unchanged for those recovered from the control and the combination group.

## Discussion

The results of this study show that CMS alone has no significant efficacy in terms of bacterial clearance either from tissue and blood or in the survival rate in the peritoneal sepsis model, even in animals infected with strains susceptible to this antimicrobial. To investigate whether these disappointing results were due to a model effect, additional experiments were made in a less severe murine pneumonia model using the strain producing OXA-48 plus CTX-M-15. Once again, the bacterial lung and blood concentrations as well as mortality were no different from those of the control group. We believe that these results are related with the development of colistin resistance during the CMS monotherapy found in the pneumonia model.

The CMS dosage used in this study has been proven to be effective in several murine experimental studies using clinical multidrug-resistant (MDR) *Acinetobacter baumannii* strains susceptible to colistin (MIC = 0.5 mg/L) (Pachon-Ibanez et al., 2010; Docobo-Perez et al., 2012; Parra Millan et al., 2016). Nevertheless, when this CMS dosage was used in a murine pneumonia model by a NDM-1-producing *K*. *pneumoniae* clinical strain, CMS monotherapy was not effective as we found in the present study (Docobo-Perez et al., 2012). Similar results were reported by de Oliveira et al. (2015) in a retrospective clinical study, observing suboptimal efficacy of polymyxins in the treatment of KPC-producing Enterobacteriaceae infections, even in combination with imipenem or meropenem.

One possible explanation to the lack of efficacy found with CMS alone, could be that the PD parameter predictive of colistin efficacy, *f*AUC_0-24_/MIC ratio (Cheah et al., 2015), has been described in infections caused by other Gram-negative bacilli, such as *Pseudomonas aeruginosa* and *A. baumannii*. So, the present results would suggest that the described *f*AUC_0-24_/MIC values to achieve various magnitudes of bacterial killing in these Gram-negative bacilli are not the appropriate for the treatment of CP-Kp infections, even for isolates with the same MIC values. However, in these studies the end-point to define the *f*AUC_0-24_/MIC values predicting colistin efficacy was to achieve a 2 log_10_ decrease in bacterial concentrations in lungs or thigh, but not to evaluate the mice survival rates.

Colistimethate sodium monotherapy did not reduce significantly the bacterial lung and blood results or the survival in the less severe pneumonia model by the strain producing OXA-48 plus CTX-M-15. However, it is noteworthy that CMS achieved a bacterial lung decrease of 2.91 log_10_ cfu/g, a value defined as optimal for *P. aeruginosa* and *A. baumannii* (Cheah et al., 2015), although the wide *SD* precluded the significance of these data. In addition, the results in the pneumonia model demonstrate that the severity of the chosen animal model is important when studying the efficacy of antimicrobial treatments. Thus, new colistin *f*AUC_0-24_/MIC values have to be defined for CP-Kp and, in our opinion, for the treatment of other Gram-negative bacilli infections.

The activity of rifampin in monotherapy in the peritoneal sepsis model was also limited, reducing only bacterial spleen concentration compared to control and CMS groups, infected with VIM-1 or OXA-48 plus CTX-M-15 strains, for which an AUC_0-24__h_/MIC = 34.5, predictor of efficacy was achieved. In the case of animals infected with the KPC-3 strain, rifampin did not decrease significantly the bacterial load in spleen or blood, but reduced the mortality. However, the survival mice conditions suggested that if the experiment were longer than 72 h, all animals included in this treatment group would have died shortly after. Moreover, a huge increase in MIC of rifampin was observed after rifampin *in vivo* monotherapy. In the less severe pneumonia model by OXA-48 plus CTX-M-15 strain, rifampin monotherapy increased significantly the bacterial clearing from lungs and blood, and also increased the mice survival. The efficacy of rifampin against other Gram-negative bacteria has been showed in experimental pneumonia models in mice (Wolff et al., 1999; Montero et al., 2004; Pachon-Ibanez et al., 2010, 2011). Nevertheless, rifampin alone cannot be an alternative due to the generation of rifampin-resistant mutants (Pachon-Ibanez et al., 2006).

Colistimethate sodium in combination with rifampin demonstrated *in vitro* a synergistic effect against the four strains, but the effect was bactericidal only against KPC-3 strain. These results are in accordance with other studies showing synergy of this combination against KPC-producing *K. pneumoniae* strains. Nastro et al. (2014) reported a synergistic effect at 24 h against 27 colistin-resistant KPC-2-producing *K. pneumoniae* clinical strains. Similarly, Elemam et al. (2010) found synergy with polymyxin B plus rifampin against 12 KPC-producing *K. pneumoniae* clinical strains. Tascini et al. (2013) also reported that CMS plus rifampin exhibited synergy against 13 colistin-resistant KPC-producing *K. pneumoniae* clinical strains, moreover being bactericidal against the 62%. On the contrary, in the case of *K. pneumoniae* producing VIM-1 the results of the present study differ from those by Tangden et al. (2014), where CMS plus rifampin did not exhibit synergy against two colistin-susceptible VIM-1-producing *K. pneumoniae* clinical strains.

With regard to the *in vivo* efficacy of CMS plus rifampin in the experimental murine peritoneal sepsis model by the colistin-susceptible VIM-1, the combination was better than the control and CMS groups taking into account the bacterial clearance from spleen and blood. When using the colistin-susceptible OXA-48 plus CTX-M-15 strain, the combination only reduced the bacterial spleen concentration. For these two colistin-susceptible strains, CMS and rifampin achieved the pharmacodynamic values described as optimal, *f*AUC_0-24_/MIC and AUC_0-24_/MIC, respectively (Jayaram et al., 2003; Gumbo et al., 2007; Landersdorfer et al., 2017). Nevertheless, is worth mentioning that the efficacy of the combination for both colistin-susceptible strains was not optimal, if we consider that mortality rates remained 93.3 and 73.3%, respectively.

Against the colistin-resistant KPC-3 strain, this combination reduced the bacterial spleen concentration and mortality, in accordance with the *in vitro* synergy studies. Nastro et al. (2014) reported a favorable outcome in five patients with colistin-resistant KPC-2-producing *K. pneumoniae* infections when treated with a combination of CMS plus rifampin. Against the other colistin-resistant strain, producing VIM-1/DHA-1, this combination was not efficacious, in contrast with the synergy observed in the time-kill studies.

In the pneumonia model caused by the OXA-48 plus CTX-M-15 strain, the activity of CMS plus rifampin was similar to that of rifampin in monotherapy, decreasing the bacterial lung concentration and increasing the mice survival compared with the control group. This combination has been successfully used against other Gram-negative bacilli (Pachon-Ibanez et al., 2010). In this pneumonia model, the strain of *K. pneumoniae* developed colistin resistance and a considerable increase in the MIC of rifampin when used as monotherapies, which was prevented with the combination. This prevention of colistin resistant mutant with this combination have been reported previously as in the *in vitro* study published by Rodriguez et al. (2010) in which they proved that the association of colistin plus rifampin was synergistic against heteroresistant *A. baumannii* isolates and prevented the development of colistin-resistant mutants (Rodriguez et al., 2010).

Limitations of the study have to be done, first that even though we wanted to evaluate the *in vitro* and *in vivo* effect of colistin plus rifampin combination against clonally unrelated clinical isolates of *K. pneumoniae* producing other carbapenemases, the number of the tested strains is low. Moreover, the less severe pneumonia model was only performed with one of the strains, and we believe it will be very interesting to do it with all of the strains.

In summary, the results obtained suggest that CMS plus rifampin has a low and heterogeneous efficacy in the treatment of severe infections, such as a peritoneal sepsis infection, caused by different CP-Kp strains, increasing only the mice survival in the infection caused by the KPC-3 strain. CMS plus rifampin combination prevents *in vivo* the development of mutants resistant to colistin. Because of the lack of available alternatives for these colistin-resistant KPC-3-producing strains, the combination of CMS and rifampin might be considered for further evaluation. Moreover, this combination showed efficacy in the less severe pneumonia model by the OXA-48 plus CTX-M-15 strain. Finally, an optimal PD index value for CMS efficacy needs to be defined for *K. pneumoniae*. Overall, the results of the present study suggest that the efficacy of CMS plus rifampin depends on the class of carbapenemase produced by *K. pneumoniae* and on the severity of the infection.

## Author Contributions

MP-I has planned and coordinated the experiments, analyzed the results, and written the manuscript. GL-H and TC-C had performed the *in vitro* and *in vivo* experiments. CD and JPP had performed the antibiotic concentrations studies by HPLC-tandem mass spectrometry (LC-MS/MS). YS had reviewed the manuscript and the experiments. JR-B, AP, and JP had reviewed the manuscript and the experiments. MCC obtained the funds to perform the studies and wrote the project, contributed to the performance of the *in vitro* experiment, and reviewed the results and the manuscript.

## Conflict of Interest Statement

The authors declare that the research was conducted in the absence of any commercial or financial relationships that could be construed as a potential conflict of interest.

## Footnotes

1 http://www.eucast.org/clinical_breakpoints/
