# Supplementary material for: Efficacy of Colistin and Its Combination With Rifampin in Vitro and in Experimental Models of Infection Caused by Carbapenemase-Producing Clinical Isolates of Klebsiella pneumoniae
Source: Front Microbiol. 2018 May 15;9:912. doi: 10.3389/fmicb.2018.00912 (PMC5962653; doi:10.3389/fmicb.2018.00912)
Supplement: Supplementary file 1 [file Data_Sheet_1.DOCX]

1. Bacterial Strains

The strains Kp07, a VIM-1 producer and the Kp21 strain, which co-produced VIM-1 and the acquired AmpC type beta-lactamase DHA-1 were isolated in a prospective multi-centre study on pAmpC- or carbapenemase-producing Enterobacteriaceae isolates from clinical samples collected for five months (February 2009-July 2009). These hospitals were distributed throughout 14 of the 18 Autonomous Communities in Spain.

1. MIRO, E., AGUERO, J., LARROSA, M. N., FERNANDEZ, A., CONEJO, M. C., BOU, G., GONZALEZ-LOPEZ, J. J., LARA, N., MARTINEZ-MARTINEZ, L., OLIVER, A., ARACIL, B., OTEO, J., PASCUAL, A., RODRIGUEZ-BANO, J., ZAMORANO, L. & NAVARRO, F. 2013. Prevalence and molecular epidemiology of acquired AmpC beta-lactamases and carbapenemases in Enterobacteriaceae isolates from 35 hospitals in Spain. Eur J Clin Microbiol Infect Dis, 32, 253-9.

The Kp28, co-producing OXA-48 and the extended spectrum beta-lactamase (ESBL) CTX-M-15, was isolated in a prospective multicenter study designed to identify Enterobacteriaceae isolates with decreased susceptibility to carbapenems. The isolates were collected from clinical infections and carriers between February and May 2013. Eighty-three Spanish hospitals from 33 out of the 50 Spanish provinces participated in the study; these 33 provinces belonged to 15 of the 17 Spanish Autonomous Communities.

1. OTEO, J., ORTEGA, A., BARTOLOME, R., BOU, G., CONEJO, C., FERNANDEZ-MARTINEZ, M., GONZALEZ-LOPEZ, J. J., MARTINEZ-GARCIA, L., MARTINEZ-MARTINEZ, L., MERINO, M., MIRO, E., MORA, M., NAVARRO, F., OLIVER, A., PASCUAL, A., RODRIGUEZ-BANO, J., RUIZ-CARRASCOSO, G., RUIZ-GARBAJOSA, P., ZAMORANO, L., BAUTISTA, V., PEREZ-VAZQUEZ, M., CAMPOS, J., GEIH, G. & REIPI 2015. Prospective multicenter study of carbapenemase-producing Enterobacteriaceae from 83 hospitals in Spain reveals high in vitro susceptibility to colistin and meropenem. *Antimicrob Agents Chemother,* 59**,** 3406-12.

The Kp29 strain, co-producing KPC-3 and the broad spectrum beta-lactamases TEM-1 and SHV-11 was isolated from an outbreak which index case was a patient with mul-tiple traumatic injuries who was transferred to the intensivecare unit (ICU) of Hospital Universitario Reina Sofía (Córdoba,Spain) from an Italian hospital in 2012. The first cases appeared in the ICU and in the surgical ward. The patient suffered a disseminatedinfection and was colonised with a carbapenem-resistant *K. pneumoniae* on 4 June 2012. The patient needed many surgical operations. Ten days after the index case infection was diagnosed, the first cases appeared in the ICU and in the surgical ward. Ten days later, additional cases, which included nosocomial pneumonia, abdominal infection, catheter-related infections, urinary tract infection and bacteraemia, appeared in many units of the hospital. During the first 8 weeks (June–July 2012), 14 patients were colonised only and 67 patients had clinical infections (50 of these being severe sepsis or septic shock). Mortality was 30% (38% for severe sepsis/septic shock).

1. LOPEZ-CERERO, L., EGEA, P., GRACIA-AHUFINGER, I., GONZALEZ-PADILLA, M., RODRIGUEZ-LOPEZ, F., RODRIGUEZ-BANO, J. & PASCUAL, A. 2014. Characterisation of the first ongoing outbreak due to KPC-3-producing Klebsiella pneumoniae (ST512) in Spain. *Int J Antimicrob Agents,* 44**,** 538-40.

2. Susceptibilities of the carbapenemase-producing *K. pneumoniae* selected strains expressed as MIC values and classification according to the clinical breakpoints definde by EUCAST^a^

| Strain | Carbapenemase (beta-lactamase co-produced) | MIC (mg/L ) [SIR]^a^ | | | | | |
| --- | --- | --- | --- | --- | --- | --- | --- |
|  |  | Fosfomycin^b^ | Meropenem | Amikacin | Gentamicin | Colistin | Rifampin |
| KPc07 | VIM-1 | 8 [S] | 2 [S] | 1 [S] | 4 [I] | 0.5 [S] | 32 [-] |
| KPc21 | VIM-1 (DHA-1) | 8 [S] | 16 [R] | 4 [S] | 2 [S] | >32 [R] | >128 [-] |
| KPc28 | OXA-48 (CTX-M-15) | 32 [S] | 2 [S] | 1 [S] | 0.5 [S] | 0.5 [S] | 32 [-] |
| KPc29 | KPC-3 | 64 [R] | >32 [R] | >32 [R] | 2 [S] | >32 [R] | 64 [-] |

^a^SIR, classification according to EUCAST definitions; S, susceptible; I, intermediate; R, resistant; -, not defined.

^b^ MIC values obtained by agar dilution method.
